# Feature Selection Method Based on High-Resolution Remote Sensing Images and the Effect of Sensitive Features on Classification Accuracy

**DOI:** 10.3390/s18072013

**Published:** 2018-06-22

**Authors:** Yi Zhou, Rui Zhang, Shixin Wang, Futao Wang

**Affiliations:** 1 Institute of Remote Sensing and Digital Earth, Chinese Academy of Sciences, Beijing 100101, China; zhouyi@radi.ac.cn (Y.Z.); zhangrui@radi.ac.cn (R.Z.); 2 University of Chinese Academy of Sciences, Beijing 100049, China

**Keywords:** SVM, feature selection, genetic algorithm, object-based, accuracy evaluation

## Abstract

With the advent of high spatial resolution remote sensing imagery, numerous image features can be utilized. Applying a reasonable feature selection approach is critical to effectively reduce feature redundancy and improve the efficiency and accuracy of classification. This paper proposes a novel feature selection approach, in which ReliefF, genetic algorithm, and support vector machine (RFGASVM) are integrated to extract buildings. We adopt the ReliefF algorithm to preliminary filter high-dimensional features in the feature database. After eliminating the sorted features, the feature subset and the C and γ parameters of support vector machine (SVM) are encoded into the chromosome of the genetic algorithm. A fitness function is constructed considering the sample identification accuracy, the number of selected features, and the feature cost. The proposed method was applied to high-resolution images obtained from different sensors, GF-2, BJ-2, and unmanned aerial vehicles (UAV). The confusion matrix, precision, recall and F1-score were applied to assess the accuracy. The results showed that the proposed method achieved feature reduction, and the overall accuracy (OA) was more than 85%, with Kappa coefficient values of 0.80, 0.83 and 0.85, respectively. The precision of each image was more than 85%. The time efficiency of the proposed method was two-fold greater than SVM with all the features. The RFGASVM method has the advantages of large feature reduction and high extraction performance and can be applied in feature selection.

## 1. Introduction

High-resolution remote sensing images are widely used in different fields, such as in land cover mapping and monitoring, classification analysis, road detection and automatic building extraction in a complex environment [1]. Extraction methods can be categorized into three main groups, namely visual interpretation, pixel and object-based methods. The main difference is the basic unit. The visual interpretation method is inefficient and easily affected by human factors. Pixel-based approaches use pixels as the basic analysis units, and object-based approaches split an image into homogeneous regions (objects) of different sizes containing multiple pixels [2]. These methods cannot satisfy the need for information extraction with an increase in image space resolution. The latter also considers the spectral, geometric, texture and topological relationships of image objects which makes it possible to utilize the contextual information.

Although the generation of hundreds of different features for each image object is an advantage of object-based approaches, the large number of features results in two main problems at the same time. On one hand, the computational burden of the procedure and the calculation of features become time-consuming. More importantly, the classification accuracy is degraded with limited samples. Thus, optimizing the feature subset is important for classification and information extraction based on high spatial resolution images [3]. Consequently, feature selection methods can be applied to tackle these problems. Feature selection methods include filter, wrapper and embedded algorithms. Filter algorithms remove features directly from the original feature set and independent of the learning algorithm, which may be a classification algorithm or a clustering algorithm. Some studies of filters methods have selected features by object-based extraction. The ReliefF (RF) is a typical feature selection algorithm, which assigns higher weights to features associated with categories, removes irrelevant features quickly and has a high operating efficiency when dealing with multiple classification problems. However, the RF algorithm has difficulty removing redundant features of datasets. Wrapper and embedded algorithms select features concurrently with the learning process and generally lead to better results than filter methods, such as genetic algorithms, which measure the performance of features with a classifier and improve the effect of the learning algorithm at the same time. Numerous object-based models for selecting features have been developed in recent decades to study optimum feature selection; these models include empirical analysis [4], separability and thresholds (SEaTH) [5], minimal redundancy maximal relevance (mRMR) [6] and the genetic algorithm (GA) [7]. Most studies have focused on one method, and only a few works have concentrated on coupling methods. S Rajesh et al. [8] proposed a method based on GA for the selection of a subset from the combination of wavelet packet statistical and Wavelet Packet Cooccurrence textural feature sets. Wang et al. [9] adopted the ReliefF algorithm (RF) to eliminate redundant features. Thus, combined with the advantages of previous methods, optimization of both the goodness-of-fit and the number of variables is worth studying.

With the development of space and sensor technology, the amount of high-resolution remote sensing data has increased dramatically [10], and features have been characterized by massiveness and high dimensionality. Thus, extracting effective features of targets from feature sets is a key stage in information extraction. Previous works have focused on single feature extraction methods and pixel-based analyses. However, another problem in objected-based methods is that primitive features are calculated for large areas. The efficiency and precision of information extraction are also challenges. They do not take advantage of the different types of feature selection and object-based methods and do not take into account the optimization of classifier parameters. 

To solve the problem of high-dimensional feature redundancy and slow convergence in objected-based information extraction, we proposed a feature extraction method integrating ReliefF, the genetic algorithm and support vector machine, namely the RFGASVM method, which combines the filter with the wrapper method. Our approach can be described in four steps: First, improved multi-scale segmentation is utilized to construct blocks of homogenous regions. Second, the features are ranked based on the weights and irrelevant features are removed; third, the preliminarily selected feature subsets are encoded with support vector machine (SVM) parameters (C and γ) into the chromosome and optimized based on the genetic algorithm (GA). Finally, the SVM classifier is employed to test the proposed method and its sensitivity is compared with related methods.

## 2. Methodology

### 2.1. Data Sets

**Figure 1:**
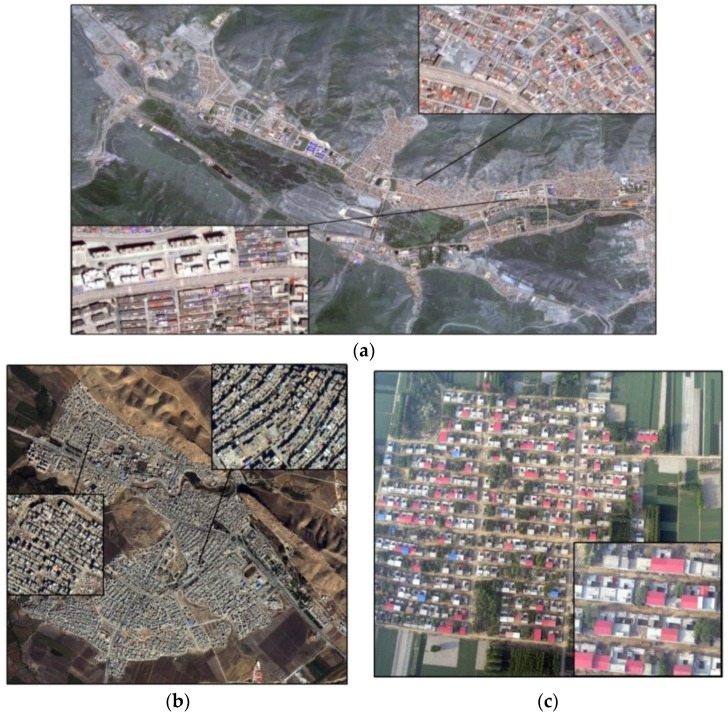
Experimental data ((**a**) GF-2 satellite data, (**b**) BJ-2 satellite data, and (**c**) unmanned aerial vehicle (UAV) data).

The described methodology was applied to high-resolution optical satellite images (1 m data for GaoFen-2 and 0.5 m data for Beijing-2) and 0.2 m unmanned aerial vehicle (UAV) images (Figure 1). The sizes of the sample images from the dataset were 900 × 500, 1000 × 1000 and 1500 × 1000, respectively. The remote sensing images included two kinds of data: panchromatic and multispectral (blue, green, red and near-infrared). For the optical remote sensing images, radiometric calibration, Gram–Schmidt pan sharpening algorithm fusion and atmospheric correction were used to obtain high spatial resolution multispectral images. For original aerial drone images, Pixel-Grid software was used to correct the difference in the original photo’s distortion and the image was rotated according to the actual overlapping direction. A position and orientation system (POS) was used under the condition of a no-control point through POS-assisted aerial triangulation after three-dimensional free network adjustment to generate an original orthographic image (DOM) from the original single photo mosaic. Although the urban remote sensing imagery used in this work was of high resolution, some object edges were still fuzzy which resulted in the object being unrecognizable from the background. Therefore, edge enhancement, an image Gaussian filtering method that reduces the effect of noise, was used. This method can also decrease the complexity of image computation and remove system noise [10]. Edge enhancement is widely used in fields such as pattern recognition and image semantic segmentation.

### 2.2. Related Theories

This research attempted to extract a building by employing object-based image analysis. The goal of extraction is to obtain the highest accuracy of identification while using relatively few features. In this work, we used the SVM classifier to extract building information. The two parameters, namely, C and γ, considerably influenced the final classification accuracy. In feature selection based on the previous GA, the number of features was barely considered and the optimization and improvement of the input parameters of the classifier were not considered. This work combined the GA and SVM classifiers using the RF feature weighting algorithm. When the fitness function in GA was set, three factors were considered (classification accuracy, number of features and feature cost). This case is a typical multi-objective optimization problem. Multi-objective optimization enables multiple targets to reach the optimal state at the same time under specific constraints.

#### 2.2.1. Multiresolution Segmentation Methods Based on High-Resolution Remote Sensing Images

We defined typical land cover elements for a segmentation according to their characteristics. An image is segmented into a cluster, called an object, and has shape information [11]. The created image objects should represent real objects [12,13]. In the present study, an adaptive multiscale segmentation model was used to create image objects and the optimization of the scale parameters. For high-resolution remote sensing images, the fractal net evolution approach (FNEA) was a regional growth algorithm from the bottom to the top. Based on the principle of least heterogeneity, the neighboring pixels with similar spectral information were merged into a homogeneous image object. All pixels that belonged to the same object after segmentation represented the same feature. In image segmentation, the spatial, spectral and shape features of the image object simultaneously operate to generate an object with spectral homogeneity and homogeneous spatial characteristics and shape features. 

**Figure 2:**
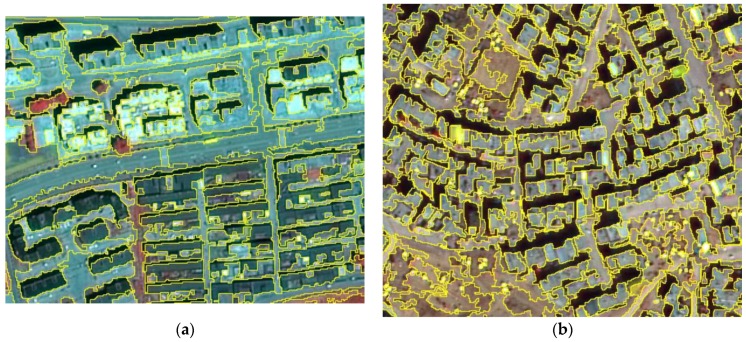

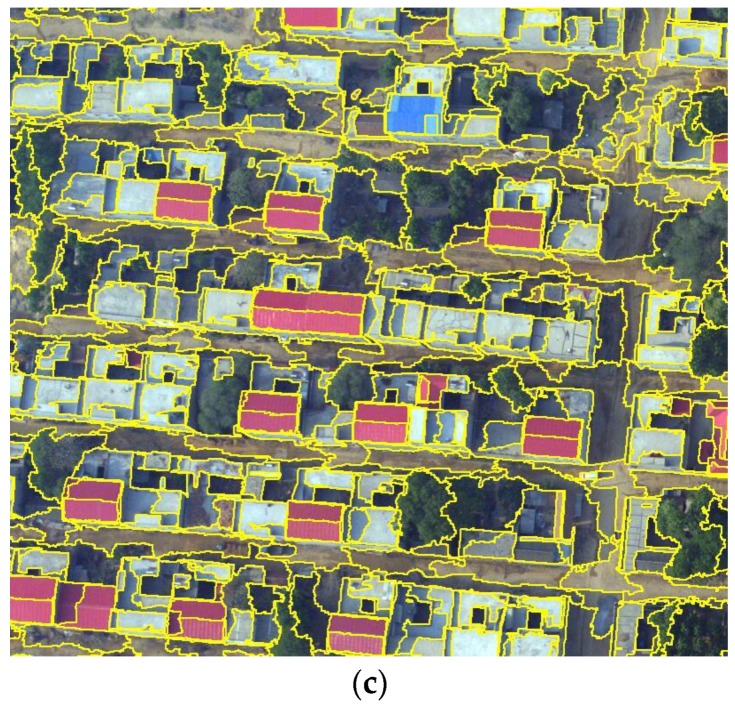
Results of segmented images ((**a**) GF-2 satellite data, (**b**) BJ-2 satellite data, and (**c**) UAV data).

The scale parameter of the FNEA segmentation algorithm was the region merger cost which was a threshold of “heterogeneity change” when the objects were merged. The multiscale expression of images was achieved to a certain extent; however, the result of previously set scaling parameters were barely recorded before segmentation. This method obtained a limited number of multiscale expressions. For issues such as unclear hierarchical relationships and scale conversion, an efficient graph-based image segmentation model (EGSM) was proposed by Felzenszwalb in 2004 [14]. This work adopted the optimal scale method, a novel bilevel scale-set model (BSM), which was proposed by Hu [15] based on EGSM. The method combines the FNEA algorithm and the layered iterative optimization of regional consolidation methods. The regional hierarchy structure was constructed, and multiscale representation of house images was obtained (Figure 2). Using the BSM, global evolutionary analysis and unsupervised scale set reduction were applied, and in processes where hierarchical region consolidation was recorded completely, the hierarchical relationships were recorded and each region was indexed on a scale. Scale reduction based on global evolution analysis was performed according to the minimum risk Bayesian decision framework. The BSM can be used to calculate the image segmentation results at any scale inversely, so as to solve the problem of adjusting the scale parameters.

#### 2.2.2. Feature Extraction Structure

In the investigated images (obtained from satellite and UAV images), the variable features were extracted using eCognition 9.1. Such features included spectral, geometry, texture, shadow, context and geoscience auxiliary features of image objects. To test the performances of feature optimization and selection, we collected a total of 113 features from high resolution remote-sensing images, which included GF-2, BJ-2 satellite images, and 67 features from UAV images. A description of the object features is shown in Table 1 and Table 2. The UAV images only contained R, G and B bands; as such, their spectral and shadow characteristics considerably differed from those of satellite images.

#### 2.2.3. Feature Selection Based on ReliefF Algorithm and Coupled GA-SVM Models

***ReliefF algorithm:*** ReliefF (RF), an extension of the Relief method, is efficient in estimating the quality of attributes but is limited to two-class problems only. This method can calculate distances between sample distributions and reliably estimate probabilities and can handle incomplete and multiclass data sets while the complexity remains the same [16,17]. When dealing with multiple types of problems, such as regression problems, for continuous data, the RF algorithm does not uniformly select the nearest neighbor sample from all different sample sets but selects the nearest neighbor sample from each set of samples; the degree of the importance of a feature is evaluated by calculating the ability to separate the nearest distance between any two classes. Given a sample set, S, sample R is selected from S, and the K nearest neighbors of sample R are found. The closest same class instance of sample R is called “near-hit (NH),” and the closest different-class instance of sample R is called “near-miss (NM).” The weight of feature 𝑡 is denoted as $\omega_{t}$, which is updated. To reduce the randomness in feature evaluation, the entire process should be repeated *m* times to obtain the average value, which is set as the final weight.

$$\omega_{t}^{i}=\omega_{t}^{i-1}+\frac{\sum_{c\neq class\left(x\right)}\frac{p\left(x\right)}{1-p\left(class\left(x\right)\right)}\sum_{j=1}^{k}diff\left(x,M\left(x\right)\right)}{m\times k}-\frac{\sum_{i=1}^{k}diff\left(x,H\left(x\right)\right)}{m\times k},$$

where *diff*() indicates the distance of the sample on feature *t*; *M*(*x*) and *H*(*x*) represent the closest same class sample and a different-class sample of sample *x*, respectively; *p*() represents the ratio of the entire samples in class $c_{i}$ to all heterogeneous samples in S; *m* is the number of iterations; and *k* is the number of nearest neighbors.

**Figure 3:**
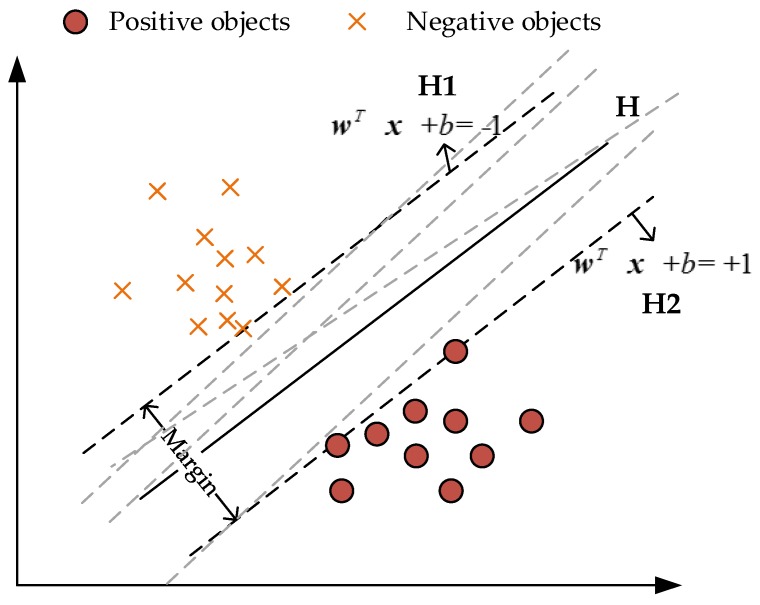
The optimal hyperplane.

***SVM model:*** The basic principles of SVM can be found in the studies of Cortes and Vapnik [18] and Devroye et al. [19]. SVM provides the optimal hyperplane (Figure 1) to maximize the margin between the closest positive and negative samples because of its effectivity in working with linearly non-separable and high dimensional datasets [20]. The white and black points are samples of two categories (Figure 3). H is the classification line, H1 and H2 represent the straight lines of the two closest samples from H and the distance between them is the classification interval. The optimal classification hyperplane makes the classification correct while maximizing the separation margin.

The original SVM algorithm seeks a linear decision surface (H) using $f\left(x\right)=w^{T}x+b$, where *w* is a dimensional coefficient vector and *b* is the offset. The linear SVM achieves an optimal hyperplane by solving the following optimization problem:

$$\min\frac{1}{2}{∥W∥}^{2}$$

$$s.t.:Y_{i}\cdot \left(W^{T}\cdot X_{i}+b\right)\geq 1\;i=1,2,3,\ldots N.$$

The optimization of the optimal hyperplane can be converted into a Lagrangian dual problem:

$$L\left(W,b,a\right)=\frac{1}{2}{∥W∥}^{2}-\sum_{i=1}^{N}a_{i}\left[\left(W\cdot X_{i}+b\right)-1\right],$$

where $a_{i}\geq 0$ and is the Lagrangian multiplier. The final classification discriminant function can be expressed as

$$f\left(X\right)=\sum_{i=1}^{N}a_{i}Y_{j}X_{i}{}^{T}\cdot X+b.$$

In most cases, SVM maps nonlinear training samples to the high-dimensional feature space and constructs linear discriminant functions. One of the most popular and frequently used kernel functions is the radial basis function (RBF), which has good generalization ability:

$$f\left(X\right)=\sum_{i=1}^{N}a_{i}Y_{j}K\left(X_{i}\cdot X\right)+b.$$

SVM uses a kernel function to map nonlinearly separable classes from a low-dimension to a higher dimension feature space. RBF is a useful function and has been implemented widely. It can map non-linear primitive features to high dimensions and deal with problems of non-linear separability. The linear kernel function is a special case of RBF. In addition, a large amount of polynomial kernel function parameters and the inner product need to be calculated. As a result, the model is complex, and there are calculation problems, such as overflow. A small number of RBF kernel function parameters are more convenient and efficient for model calculating. An RBF kernel needs two parameters (C and γ) which should be set to obtain an improved classification model. C is a preset value that penalizes the misclassification, and γ controls the width of the RBF kernel [21]. To obtain an optimal combination of C and γ, the present work used grid search and 10-fold cross-validation. Grid search is a process where various combinations of C and γ are selected within a predefined range at a certain interval. Cross-validation is used to test the accuracy of classification in terms of different combinations of C and γ.

***GA:*** This algorithm consists a series of genetic operations, such as selection and crossover, which are mutations, to generate a new generation of groups which are gradually evolved to be included or become close to the optimal solution [22]. In feature selection, first, the feature set to be optimized and C and γ in the SVM classifier are encoded into a chromosome. A fitness function is constructed considering the recognition accuracy of the house, and an initial population is generated. The initial population is selected through selection and cross-mutation operations. Individuals in the population are optimized to produce the optimal subset of features and the optimal C and γ.

**Figure 4:**
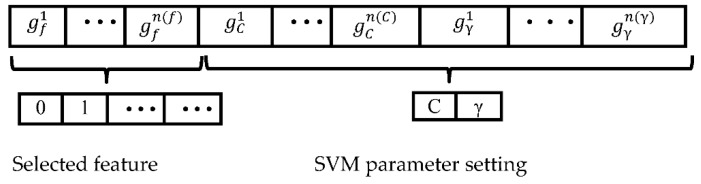
The chromosome design. SVM: support vector machine.

The basic procedure for chromosome coding can be summarized as shown in Figure 4. In chromosome design, the chromosome includes three parts: the feature set, C and γ. The detailed design for the chromosome is shown in Figure 3, where $g_{f}^{1}$ to $g_{f}^{n\left(f\right)}$ is the encoding of feature set (*f*), n(f) represents the bits of the code, in which n represents a sequence of numbers, 1 represents that the feature is selected and 0 represents that the feature is ignored; $g_{C}^{1}$ to $g_{C}^{n\left(C\right)}$ encodes the SVM parameter C, and $g_{\gamma }^{1}$ to $g_{\gamma }^{n\left(\gamma \right)}$ encodes the SVM parameter γ, while n(C) and n(γ) represent the bits of the code.

Based on the feature weights calculated by RF, the initial population is provided for GA. In the population initialization phase, the population size of GA is based on feature weights and the maximum number of iterations must be set appropriately. The weights of the retained features are normalized, and the result is set as the probability that the feature is selected. An individual’s fitness of the proposed method is primarily determined by three evaluation criteria, namely the classification accuracy, the size of the selected feature subset and the feature cost. However, the feature cost was ignored in previous studies. Thus, small feature subsets include a low total feature cost and a high classification accuracy, in which the optimal individual (single optimal feature) demonstrating good fitness is chosen during the evolutionary process; an individual’s fitness can be obtained as follows:

$$\mathrm{fitness}=W_{a}\times \mathrm{accuracy}+W_{f}\times \left(1-\frac{1}{n}\sum_{i=1}^{n}f_{i}\times C_{i}\right),$$

where, $W_{a}$ represents the weight of the classification accuracy, which is the classification results of the test samples. $W_{f}$ represents the weight of the number of selected features with feature costs, and $C_{i}$ represents the cost of the features. If $f_{i}=1$, then the feature is selected as an input feature for the SVM classifier; if $f_{i}=0$, then the feature is ignored. Algorithm 1 shows the flow of the proposed feature selection method.

**Algorithm 1.** Flow of the proposed feature selection method.**Input** S is an initial sample feature set and $g_{f}^{n\left(f\right)}$, $g_{C}^{n\left(C\right)}$, $g_{\gamma }^{n\left(\gamma \right)}$ are the initial population, where f encodes the feature set, C and γ are the encoded SVM parameters.**Output** Extracted features based on the RFGASVM method
**Repeat**
Sequence sample feature set using ReliefF and the weight of feature 𝑡 ($\omega_{t}^{i}$) is updated m times to obtain the average valuePopulation initialization with the RFGA methodSet the individual’s fitness. The feature cost is $\frac{1}{n}\sum_{i=1}^{n}f_{i}\times C_{i}$, where $C_{i}$ represents the cost of features and $f_{i}=1$, 0
**Until the** termination test is met4.A small feature subset with a low total feature cost and high classification accuracy

(Remark: RFGASVM: ReliefF, genetic algorithm, and support vector machine method; RFGA: integrate ReliefF with genetic algorithm method).

**Figure 5:**
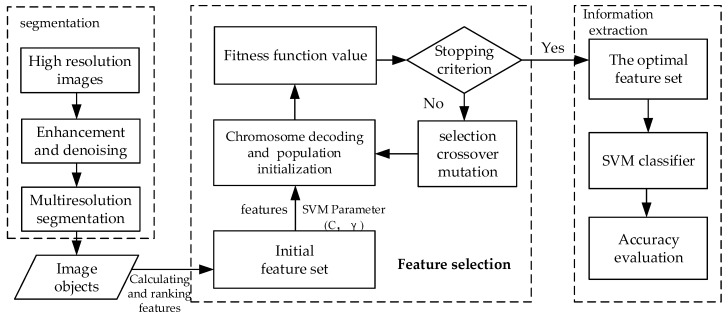
Flow chart of the extraction of house information based on the RFGASVM optimization algorithm.

The algorithm mentioned above is shown in Figure 5.

### 2.3. Accuracy Assessment

The accuracy assessment was conducted using a confusion matrix from the perspective of classification, and the performance of the SVM classifier was evaluated by *precision, recall,* and *F1-score* based on recognition rate. We evaluated the accuracy of the proposed method from these two perspectives.

From the perspective of classification, *the overall accuracy* (*OA*), *the producer’s accuracy* (*PA*), *the user’s accuracy* (*UA*), and *the Kappa coefficient* (*Kappa*) [23] were evaluated using the accuracy evaluation function of eCognition. *The Kappa coefficient* is the most significant one, because it marks the robustness of an algorithm. If the coefficient is over 0.6, the algorithm is recognized as having good performance. *OA* is an overall assessment which indicates the general performance of the technique.

$$OA=\frac{TP+TN}{T},$$

$$Kappa=\frac{T\times \left(TP+TN\right)-\sum }{T\times T-\sum },$$

$$PA=\frac{TP}{TP+FN},$$

$$UA=\frac{TP}{TP+FP},$$

where ∑=(TP+FP)×(TP+FN)+(FN+TN)×(FP+TN). *TP* is the correctly extracted pixels; *FP* is the incorrectly extracted pixels; *TN* is the non-building pixels that are correctly rejected; *FN* is the building pixels that are not detected.

From the perspective of the recognition rate [24], *precision* is the percentage of building objects that are correctly classified by the SVM classifier. *Recall* is the percentage of correctly classified building objects among all actual buildings. The *F1-score* is a combination of precision and recall:

$$Pre=\frac{Ntp}{Ntp+Nfp}\times 100\%,$$

$$Rec=\frac{Ntp}{Ntp+Nfn}\times 100\%,$$

$$F1=2\times \frac{Pre\times Rec}{Pre+Rec}.$$

The building extraction can be considered to be a binary classification, where building objects are positives and the remaining non-building objects are negatives. *Ntp* denotes the number of buildings correctly extracted; the detected buildings are at least partially real. *Nfp* denotes the number of buildings mistakenly extracted. *Nfn* denotes the number of non-buildings mistakenly extracted.

## 3. Experimental Results and Discussion

### 3.1. Selection of Building Samples

**Figure 6:**
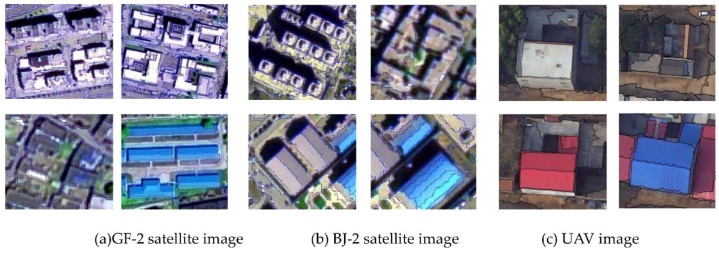
Schematic of training and test samples.

According to the characteristics of different sensor images and the principle that human eyes can recognize houses in high-resolution images, a remote sensing classification system for houses was determined. The houses were divided into four types: high-rise buildings, multistorey buildings, factory buildings and general houses. Various typical house samples were selected through the segmentation of GF-2, BJ-2 and UAV images. During sample selection, the samples were distributed as evenly as possible and included each type of house. Given that the SVM model was used, roads, vegetation, shadows, water and bare land should have been selected. To reduce the influence of mixed pixels on classification accuracy, we tried to avoid mixed pixels when selecting a sample. The house training samples and test samples are shown in Figure 6 and the selected land types and their quantities are listed in Table 3.

### 3.2. Building Identification Results and Accuracy of the Proposed Method

**Figure 7:**
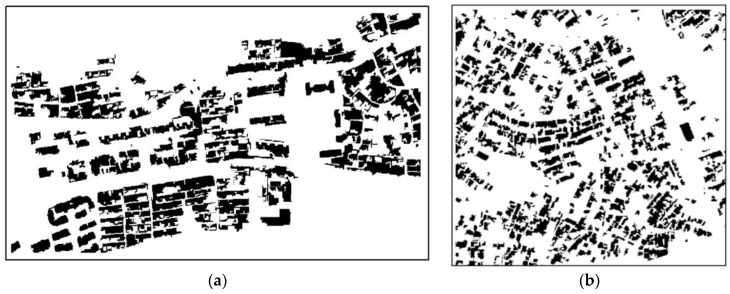

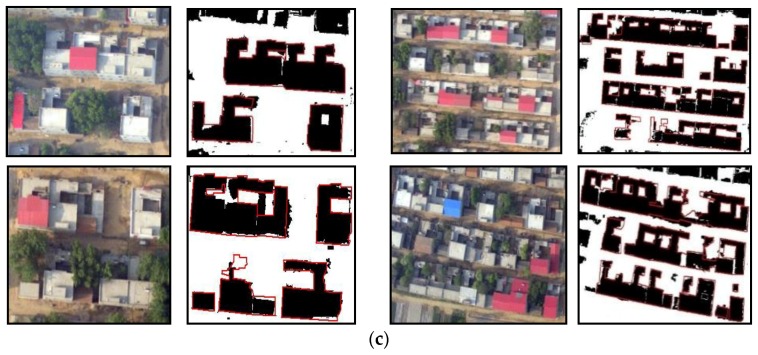
Extraction results of urban and rural areas.

The new method was validated with three images captured by the GF-2 satellite, BJ-2 satellite, and UAV. These images described parts of urban and rural areas. However, visual interpretation on the image of the entire administrative area is rarely practical; as such, three typical images, which contained dark roofs and similar spectral characteristics with roads, were selected to obtain reliable results on performance. Therefore, the performances of our feature optimization algorithm may not seem the same as those in other studies. However, the proposed method acquired satisfactory results under relatively poor conditions, and high accuracy would be easily accessible. The experiments were performed 15 times on the three resolution images (Figure 7a: GF-2; Figure 7b: BJ-2; Figure 7c: UAV), and the average value represents the highest recognition accuracy. Figure 7a shows the housing extraction results of GF-2 imagery; in the figure, buildings are differentiated from other land types, especially high-rise and multistorey buildings in urban areas. Figure 7b was the most difficult to detect among the three images, because all the buildings and roads shared similar spectral characteristics. It is difficult to distinguish buildings from the background when there is no shadow from the building. We obtained four different rural houses in the UAV remote sensing images and compared the proposed algorithm with manual visual interpretation. The experimental results are shown in Figure 7c. The left image is the original remote sensing image; the black area on the right represents the results of the proposed algorithm, and the red polygons represent the visual interpretation results.

For the optimal accuracy of building identification, the selected feature subset of image 1 (GF-2) contains the mean b, mean r, SBI, GLCM mean (all direction), MBI, NDVI, length/width and the elliptic fit; image 2 (BJ-2) contains the max.diff, mean r, shape index, GLCM homogeneity (all directions), GLDV entropy and Chen3; image 3 (UAV) contains the ratio g, green index, brightness, rectangular Fit, density, GLCM SD, GLCM ASM, main direction, length/width and the elliptic fit. The stastics analysis of the accuracy is shown in Table 4. The new technique has a robust kappa coefficient, concentrated at 0.83. The preferred features are more robust and resist variation in the images, whether their buildings are densely distributed or not. The UAV images only have R, G, and B bands; as such, the longer the optimization time for extracting classification features is, the higher the number of features used for identification is.

### 3.3. Verification of Feature Selection Based on Kernel Density Estimation

**Figure 8:**
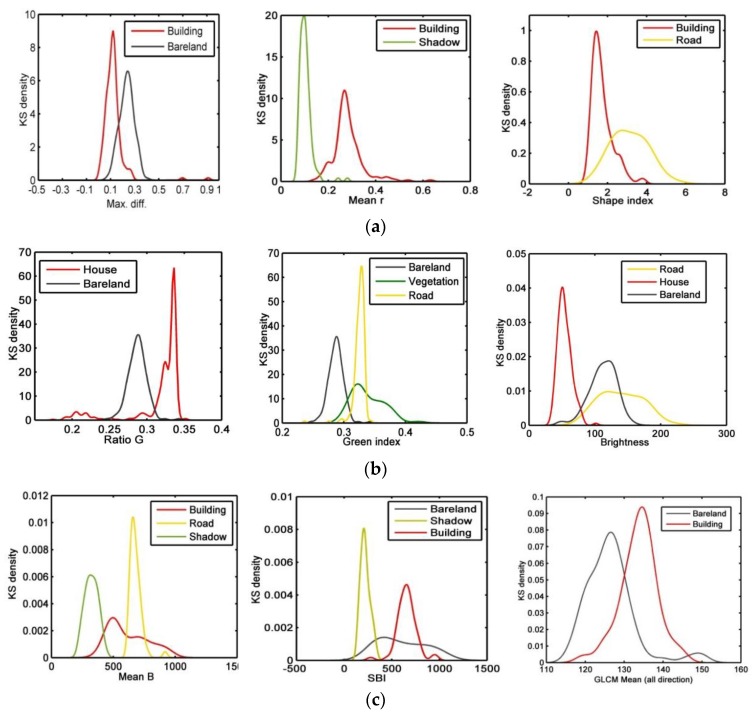
Probability distribution density of different object features based on extraction from high-resolution images ((**a**) BJ-2 imagery, (**b**) UAV imagery, and (**c**) GF-2 imagery).

To analyze the object features, we used the KS density (Kernel Smoothing function estimate) to fit the probability distribution density of every feature for different category samples. The kernel distribution is a nonparametric representation of the probability density function (PDF) of a random variable. Fitting the probability distribution of an object feature by using the KS density is reasonable. The formula of the kernel density estimator is as follows [25]:

$$\hat{f_{h}}\left(x\right)=\frac{1}{nh}a_{i=1}^{n}K\left(\frac{x-x_{i}}{h}\right),$$

where *n* is the sample size; *x_i_* is the object feature value; *K*(.) is the Kernel Smoothing function; and *h* is the bandwidth. The kernel distribution places the values into discrete bins and sums the component smoothing functions for each data value to produce a smooth, continuous probability curve. Figure 8 below represents the probability distribution density of different object features from the three typical study areas. Land types can be well distinguished based on the features, and residential land can be separated from other adjacent land types, thereby facilitating information extraction.

### 3.4. Accuracy and Efficiency Assessment of Selected Features

We compared the RFGASVM method with other methods, namely, SVM with all features and RFSVM without GA, to optimize parameters. The OA values of RFGASVM, SVM (all features) and RFSVM are shown in Table 5. The OA for features selected by RFGASVM had a mean value of over 80%, and UAV imagery reached 91.3%. This finding indicates that features selected by RFGASVM are more representative than those selected by the other two methods. The accuracy of SVM (all features) also reached 80%; however, the use of many features brings huge computational costs. RFSVM had a lower accuracy than the other two methods. RFGASVM-selected features achieved higher accuracy and effectiveness based on the OA, Kappa coefficient and feature number. Hence, the proposed method is more suitable for the identification of residential land. Table 6 shows that our feature dimensionality reduction and optimization strategy outperformed other methods for high-resolution remote sensing images. The precision of each image was more than 85%, and the precision and recall were significantly greater than the other two methods.

**Figure 9:**
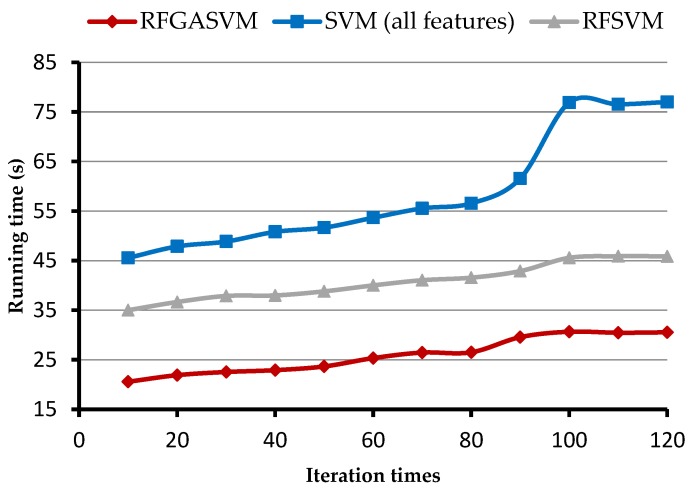
Comparison of the efficiency of proposed RFGASVM and related methods using different iteration times.

Feature redundancy increases the size of the search space and affects the speed of algorithms. We extracted the running time from different iteration times of BJ-2 imagery and later, compared the proposed method with the SVM (all features) and RFSVM without GA to measure the computational efficiency. As shown in Figure 9, the SVM (all features) method took more time using a large number of features. This is because global optimization takes a lot of time with increased iterations. The implementations of the RFGASVM took much less time—up to two times faster than the other two methods. The results show that efficiency is greatly improved when dealing with images of large regions.

## 4. Conclusions and Future Work

In this study, we proposed a novel feature dimensionality reduction and optimization strategy to extract buildings using an object-based image analysis approach. The feature selection method is based on the ReliefF, genetic algorithm (GA) and support vector machine (SVM) methods and is called the “RFGASVM” method. We collected several samples using results from three high-resolution remote sensing images (GaoFen-2, Beijing-2 and UAV images), and then selected features to extract buildings to evaluate the performance of the proposed method. The approach consisted of four steps: First, the image pixels from the image were grouped using a multiresolution segmentation algorithm to form objects. Then, features were calculated by object-based image analysis, and stable features were derived from the inherent characteristics of objects and were given the possibility of being implemented on high-resolution images. Features were ranked based on ReliefF method to reduce the redundancy. The preliminarily selected feature subset and SVM parameters were optimized based on the genetic algorithm (GA) by selecting the optimal feature sets from the remaining sorted features. Finally, the experimental results demonstrated the effectiveness of the proposed method in terms of the efficiency and classification accuracy. 

The proposed feature selection method reduces the redundancy for object-based image analysis and is well-suited for high-resolution remote sensing images. In addition, it can be applied to feature selection and information extraction and has the advantage of a higher reduction rate. In our future research, we plan to design and implement high-quality samples for high-performance feature selection.

## Figures and Tables

**Table 1:** Description of features extracted from high resolution remote-sensing images.

Feature Name	Feature Description
**Spectral features**	Mean L (R, G, B, NIR); brightness; SD L (R, G, B, NIR); ratio L (R, G, B, NIR); max.diff; MBI index (Huang Xin et al.); BAI:(B − NIR)/(B + NIR); NDBI: (MIR − NIR)/(MIR + NIR); NDVI: (NIR − R)/(NIR + R); DVI: NIR − R; RVI: NIR/R; SAVI: 1.5 × (NIR − R)/NIR + R + 0.5); OSAVI: (NIR − R)/(NIR + R + 0.16); SBI: (R^2^ + NIR^2^)^0.5^; NDWI:(G − NIR)/(G + NIR)
**Geometrical features**	Area; length; width; length/width; boundary length; pixel number; shape index; density; main direction; asymmetry; compactness; rectangular fit; elliptic fit; differential of morphological profiles (DMP)
**Textural features**	GLCM entropy; GLCM angular second moment; GLCM correlation; GLCM homogeneity; GLCM contrast; GLCM mean; GLCM SD; GLCM dissimilarity; GLDV angular second moment; GLDV entropy; GLDV contrast; GLDV mean
**Shadow indexs**	SI:(R + G + B + NIR)/4; Index related to shadow: Chen1: 0.5 × (G + NIR)/R − 1; Chen2: (G − R)/(R + NIR); Chen3: (G + NIR − 2R)/(G + NIR + 2R); Chen4: (R + B)/(G − 2); Chen5: |R + G − 2B|
**Contextual features**	Object numbers; object layers; image resolution; mean of image layers
**Geo-Auxiliary features**	Digital elevation model(DEM); slope; aspect; building vectors

(Remark: Mean L: mean of the bands; SD L: Standard Deviation of the bands; ratio L: ratio of the bands; MBI: Morphological Building Index; BAI: Building Area Index; NDBI: Normalized Difference Build-up Index; NDVI: Normalized Difference Vegetation Index; DVI: Difference Vegetation Index; RVI: Ratio Vegetation Index; SAVI: Soil-Adjusted Vegetation Index; OSAVI: Optimized Soil Adjusted Vegetation Index; SBI: Soil brightness index; NDWI: Normalized Difference Water Index; GLCM: Gray Level Co-occurrence Matrix; GLDV: Grey Level Difference Vector; SI: Shadow Index; Chen: Custom features).

**Table 2:** Description of features extracted from unmanned aerial vehicle image.

Feature Name	Feature Description
**Spectral features**	Mean L (R, G, B, NIR); brightness; SD L (R, G, B, NIR); ratio L (R, G, B, NIR); max.diff; Green Index: GR = G/(R + G + B); Red-Green Vegetation Index: NGRDI = (G − R)/(G + R); GLI = (2G − R − B)/(2G + R + B)
**Geometrical features**	Area; length; width; length/width; boundary length; pixel number; shape index; density; main direction; asymmetry; compactness; rectangular fit; elliptic fit; differential of morphological profiles (DMP); digital surface model(nDSM); height standard deviation
**Textural features**	GLCM entropy; GLCM angular second moment; GLCM correlation; GLCM homogeneity; GLCM contrast; GLCM mean; GLCM SD; GLCM dissimilarity; GLDV angular second moment; GLDV entropy; GLDV contrast; GLDV mean
**Shadow indexs**	Chen4: (R + B)/(G − 2); Chen5: |R + G − 2B|
**Contextual features**	Object numbers; object layers; image resolution; mean of image layers
**Geo-Auxiliary features**	Digital elevation model(DEM); slope; aspect; building vectors

(Remark: GR: Green Index; GLR: Green Leaf Index; NGRDI: Red-Green vegetation index).

**Table 3:** Sample statistics selected from different high-resolution images.

Data	Sample Category	Building	Road	Vegetation	Shadow	Water	Bare Land
GF-2 image	Training samples Testing samples	95 106	75 92	85 113	68 72	70 85	92 110
BJ-2 image	Training samples Testing samples	95 102	80 95	87 92	79 86	-- --	91 95
UAV image	Training samples Testing samples	105 112	110 115	95 102	90 98	-- --	90 102

**Table 4:** Statistical analysis of the accuracy of proposed method for processing high-resolution imagery.

High-Resolution Imagery	GF-2 Satellite Image	BJ-2 Satellite Image	UAV Image
Overall accuracy (OA)	88.52	89.75	91.3
Kappa coefficient	0.8	0.83	0.85
Producer’s Accuracy (PA)	91	93.12	96.21
User’s Accuracy (UA)	89.65	89	90.38
Number of features	8	6	10
Optimization time	7.85	13.79	18

**Table 5:** Comparison among RFGASVM and related methods.

Experimental Data	Evaluation Index	RFGASVM	SVM (All Features)	RFSVM
**GF-2 imagery**	Overall accuracy (OA) Kappa coefficient Number of features	88.52 0.90 8	86.46 0.88 85	83.02 0.85 13
**BJ-2 imagery**	Overall accuracy (OA) Kappa coefficient Number of features	89.75 0.93 6	81.06 0.85 85	80 0.90 13
**UAV imagery**	Overall accuracy (OA) Kappa coefficient Number of features	91.30 0.91 10	86 0.88 70	90.25 0.85 15

**Table 6:** Results of the test samples of satellite and UAV imagery between proposed RFGASVM and related methods in terms of precision, recall and F1-score.

Experimental Data	Method	Precision	Recall	F1-Score
**GF-2 imagery**	**RFGASVM** SVM (all features) RFSVM	85.50 83.25 81.0	86.81 82.53 80.0	86.15 82.89 80.50
**BJ-2 imagery**	**RFGASVM** SVM (all features) RFSVM	89.51 78.67 80.10	88.12 77.50 79.1	88.81 78.08 79.60
**UAV imagery**	**RFGASVM** SVM (all features) RFSVM	92.25 86.51 87.35	90.05 78.81 85.0	91.14 82.48 86.41
